# A 17-level octuple boost switched-capacitor inverter with lower voltage stress on devices

**DOI:** 10.1038/s41598-024-65211-0

**Published:** 2024-06-22

**Authors:** Majid Hosseinpour, Meysam Noori, Mahdi Shahparasti

**Affiliations:** 1 Department of Electrical Engineering, University of Mohaghegh Ardabili, Ardabil, Iran; 2 School of Technology and Innovations, University of Vaasa, Vaasa, Finland

**Keywords:** Multilevel inverter, Switched-capacitor, Lower voltage stress of devices, Inrush current, Energy science and technology, Renewable energy

## Abstract

This paper presents a new structure for switched-capacitor multilevel inverter with octuple voltage gain capability. The proposed inverter utilizes three capacitors, 13 semiconductor switches, three diodes, and an input voltage source to achieve a 17-level output voltage. The switched capacitors naturally achieve voltage balancing without the need for sensors or additional circuits, indicating the ease of control of the proposed structure. To control the inrush current of the switched capacitors, a charge limiting inductor has been utilized in the charging path of the capacitors. This not only reduces the inrush current of the capacitors and the input source current but also enables faster capacitor charging and extends their lifetime. The switches used in the proposed structure can withstand a maximum of 4 times the input voltage value or the half of the maximum output voltage, which is a significant advantage for the proposed structure. A detailed comparison with similar structures is provided to examine the advantages and disadvantages of the suggested inverter. The procedure of self-voltage balancing of the capacitors and the functional modes of the proposed topology has been explained in detail. The proposed structure is suitable for applications such as renewable energy sources transfer to load or grid. The performance of the proposed topology under different conditions is confirmed through simulation in the Matlab\Simulink software and the implementation of the laboratory sample.

## Introduction

Due to the importance of converting DC to AC in connecting renewable energy sources to the power grid, multilevel inverters (MLIs) have received considerable attention as one of the most advanced and efficient technologies in this field. One of the reasons for the popularity of multilevel inverters is that they have features such as better power quality, modularity, lower voltage stress on devices, better harmonic distortion, and fault tolerance^1–4^. The traditional constructions of multilevel inverters, known as a neutral point clamped (NPC), flying capacitor (FC), and cascaded H-bridge (CHB), are utilized to replace traditional two-level and three-level inverters in applications such as solar systems, electric vehicles, electric drives, etc.^5–7^. With the increase of output voltage levels in conventional structures, the device number has risen, and problems such as control complexity arise. Besides, the generated voltage magnitude of the renewable energy resouces such as solar arrayes is not high enough to interconnect to the grid or load directly. Hence, a DC-DC converter will be necessary to boost the output voltage range of the inverter. Reference^8^ provides a method to reduce the number of input sources for multilevel inverters. In this structure, the voltage boost factor is increased using capacitors and one input source. The main challenge in this structure is how to balance the voltage of the capacitors since the voltage of the utilized capacitors cannot be automatically balanced.

Researchers have introduced switched-capacitor based MLIs with self-balancing capability to plummet the complexity of the control and the cost of the inverter for renewable energy sources. The large number of switches and the high charge current of the switched capacitors are the main challenges of these structures^9^. A charging current during the charging of the capacitor is generated through the input voltage source so that the switches placed in the charging path must bear such a high charging current. This high charging current reduces the efficiency of the converter and the lifetime of its devices. In^10^, a procedure to solve this problem is provided which the inrush currents are limited by utilizing a current limiting inductor. In^11^, a quasi-impedance source converter has been utilized to charge the capacitors instead of the power source, which, in addition to reducing the inrush charging current of the capacitors, has led to an increase in the voltage gain as well. In^12^, a soft switched DC-DC converter interconnects the input voltage source to the switched capacitors. This procedure draws a continuous and controlled current from the input source and alleviates the inrush charging current of the switched capacitors. The topology presented in^13^ employs an H-bridge module to generate bipolar voltage levels, in which four switches must withstand the output peak voltage. In this structure, there is no method to control the inrush charging current of the switched capacitors.

The interest of researchers in the field of switched-capacitor multilevel inverters, is designing single-source structure utilizing fewer semiconductor devices with lower voltage stress as much as possible. By reducing the voltage stress of the inverter and dividing it between the devices, it is possible to use the switches with lower ratings. Reducing the rating of switches results in a lower total cost of the inverter. A 17-level quadruple boost switched-capacitor structure is introduced in^14^, employing 12 switches, 2 diodes, and 3 capacitors. Although this structure is independent of the H-bridge module in producing negative voltage levels, still three switches must withstand the maximum output voltage. The structure presented in^15^ creates a 17-level waveform utilizing 12 switches, 5 diodes, and 4 capacitors and has an eight-fold voltage gain. In this structure, the maximum voltage stress of the switches is half of the output voltage for eight switches. The structure presented in^16^ utilizing an input voltage source, twenty switches, and four capacitors can increase the voltage four times and produce bipolar voltage levels without needing an H-bridge. In this structure, the maximum voltage stress of the switches is 50% of the output voltage for twelve switches. The topology presented in^17^ utilizes 15 switches and 3 capacitors to generate a 13-level output voltage with sixtuple boost capability. In this structure, the voltage stress is divided between the switches, and as a result, they bear less stress. In this structure, the maximum voltage stress on the switches is half of the output voltage for four switches, and for other switches is below the half.

In this paper, a 17-level switched-capacitor structure is presented, which can increase the output voltage up to 8 times the input voltage. The advantages of the proposed structure are included as follows:

- Use of a single input voltage source
- Octuple boost factor
- Generation of positive and negative voltage levels without the need for an H-bridge module
- Lower voltage stress of switches, lower losses and lower cost
- Self-balancing capability for voltage of capacitors
- Capability to perform in inductive loads
- Reducing inrush currents in capacitors using a charge current limiting inductor

In the following, the structure of the paper is explained as follows: The proposed structure is introduced in section two, then the principles of operation, switching modes, and charging and discharging of capacitors are described in detail. In the following, the method of calculating and designing the utilized capacitors in the proposed structure is illustrated. In the third section, the losses of the proposed converter have been evaluated and, a comparative evaluation has been carried out in section four. The results of the simulation and implementation of the laboratory setup are presented in the fifth section. In the sixth section, conclusions and summaries are made.

## Proposed structure

**Figure 1:**
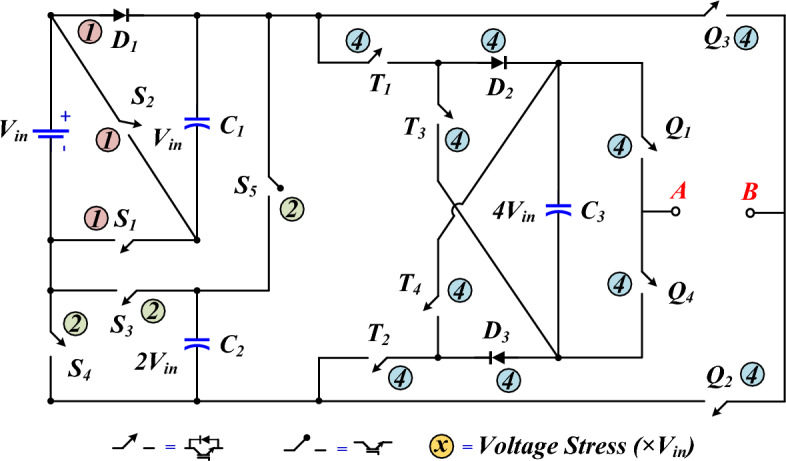
Proposed 17L SCMLI topology.

The proposed 17-level inverter circuit is displayed in Fig. 1. According to this figure, the proposed structure consists of a DC power supply (*V*_*in*_), 13 switches, 3 diodes, and 3 capacitors. The rated voltage of capacitors *C*_*1*_, *C*_*2*_, and *C*_*3*_ are *V*_*in*_, 2*V*_*in*_, and 4*V*_*in*_, respectively. An inductor paralleled with a diode in series with the input voltage source has been utilized to control the inrush charging current of the capacitors. In addition to controlling the charging current of the capacitor, this also alleviates the input current peak. The proposed structure does not need an H-bridge module to generate bipolar voltage levels, and none of the power switches withstand the maximum output voltage.

**Figure 2:**
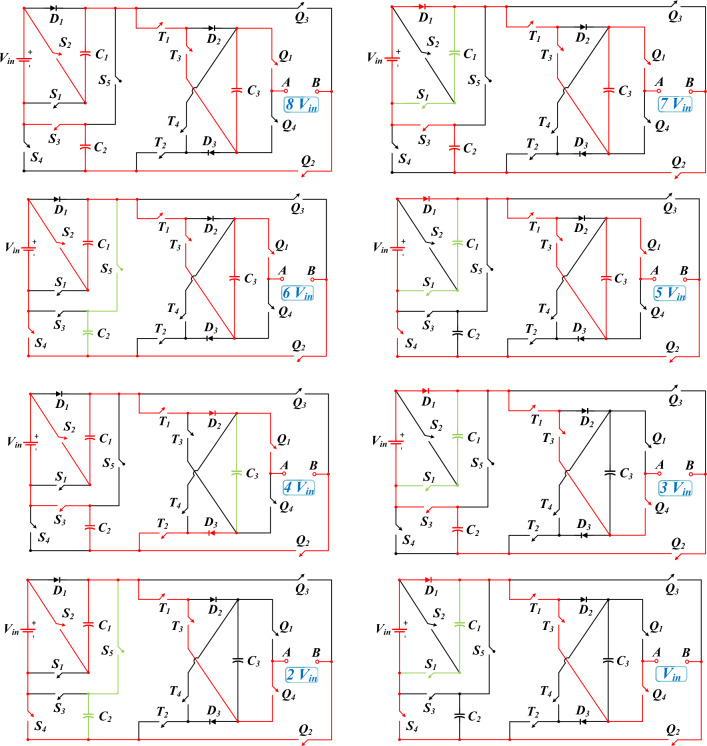
Current flow paths to generate different voltage levels in the positive half-cycle.

Figure 2 demonstrates the paths of current passing and how capacitors are charged and discharged to generate different voltage levels. Table 1 reveals the power switches that produce each voltage level. In this Table, the on and off states for the switches are indicated by 1 and 0, respectively. Besides, C means charging, D means discharging, and—means no change in capacitor voltage.

**Table 1:** Switching modes of the proposed structure.

S_1_	S_2_	S_3_	S_4_	S_5_	T_1_	T_2_	T_3_	T_4_	Q_1_	Q_2_	Q_3_	Q_4_	C_1_	C_2_	C_3_	V_O_
0	1	1	0	0	1	0	1	0	1	1	0	0	D	D	D	8V_in_
1	0	1	0	0	1	0	1	0	1	1	0	0	C	D	D	7V_in_
0	1	0	1	1	1	0	1	0	1	1	0	0	D	C	D	6V_in_
1	0	0	1	0	1	0	1	0	1	1	0	0	C	–	D	5V_in_
0	1	1	0	0	1	1	0	0	1	1	0	0	D	D	C	4V_in_
1	0	1	0	0	1	0	1	0	0	1	0	1	C	D	–	3V_in_
0	1	0	1	1	1	0	1	0	0	1	0	1	D	C	–	2V_in_
1	0	0	1	0	1	0	1	0	0	1	0	1	C	–	–	V_in_
0	1	1	0	0	1	1	0	0	0	1	0	1	D	D	C	0
1	0	0	0	0	1	0	0	0	1	0	1	0	C	–	–	0
1	0	0	1	0	0	1	0	1	1	0	1	0	C	–	–	−V_in_
0	1	0	1	1	0	1	0	1	1	0	1	0	D	C	–	−2V_in_
1	0	1	0	0	0	1	0	1	1	0	1	0	C	D	–	−3V_in_
0	1	1	0	0	1	1	0	0	0	0	1	1	D	D	C	−4V_in_
1	0	0	1	0	0	1	0	1	0	0	1	1	C	–	D	−5V_in_
0	1	0	1	1	0	1	0	1	0	0	1	1	D	C	D	−6V_in_
1	0	1	0	0	0	1	0	1	0	0	1	1	C	D	D	−7V_in_
0	1	1	0	0	0	1	0	1	0	0	1	1	D	D	D	−8V_in_

According to Fig. 2, it is clear that to generate different voltage levels, 6 power electronic switches are placed in the path of the load current. Meanwhile, in most levels, another power switch is turned on to charge the capacitors as well. In most 17-level structures, the number of conducting power switches in the path of the load current or capacitor charging is more than this number. This point will decrease the conduction losses of the proposed structure, which will be studied in the relevant section.

### Capacitor calculations

**Figure 3:**
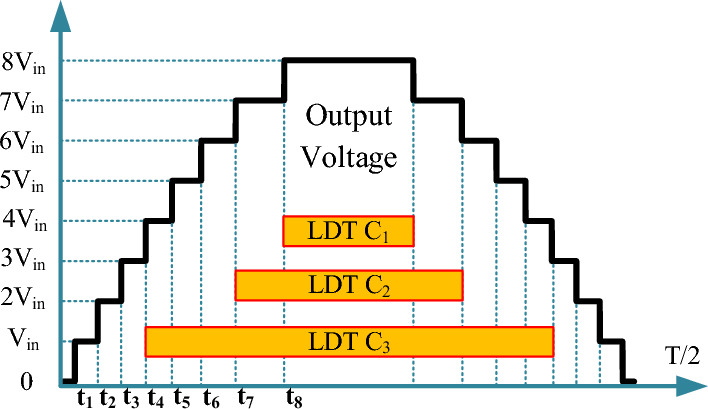
The method of discharging capacitors at different voltage levels.

In switched-capacitor multi-level inverters, self balancing of the capacitors’ voltage is provided by their charging and discharging using parallel and series connection with the input DC voltage source, respectively. Choosing the suitable capacitor capacity has a significant influence on reducing capacitor voltage ripple and depends on various factors such as the longest discharging time (LDT) and loading conditions^18^. By reducing the voltage ripple of the capacitor, the losses of the converter are decreased, and the quality of the output voltage waveform is enhanced. Figure 3 displays how capacitors are discharged to create different output voltage levels. The capacitor *C*_*1*_, which is fixed at the voltage of *V*_*in*_, is discharged at the voltage levels of 2*V*_*in*_, 4*V*_*in*_, 6*V*_*in*_, and 8*V*_*in*_, and charged at the voltage levels of *V*_*in*_, 3*V*_*in*_, 5*V*_*in*_, and 7*V*_*in*_. The capacitor *C*_*2*_, which is fixed at the voltage of 2*V*_*in*_, is charged at the voltage levels of 2*V*_*in*_, and 6*V*_*in*_, and charged at the voltage levels of 3*V*_*in*_, 4*V*_*in*_, 7*V*_*in*_, and 8*V*_*in*_. Besides, the capacitor *C*_*3*_, which is fixed at the voltage of 4*V*_*in*_, is discharged at 5*V*_*in*_ to 8*V*_*in*_, and charged at the voltage level of 4*V*_*in*_. Considering the longest discharging intervals, the capacity of *C*_*1*_, and *C*_*2*_ will be suitable values, and only the capacity of *C*_*3*_ is the main challenge of the proposed structure.

The discharge amount of capacitors is calculated by Eq. (1), in which *I*_*P*_ is the peak value of the output current, *t*_*a*_ and *t*_*b*_ are the initial and final moments of the corresponding capacitor discharge, and *f*_*o*_ is the output frequency.

1 $$ Q_{C} = \int_{{t_{a} }}^{{t_{b} }} {I_{P} } \sin \left( {2\pi f_{o} t} \right)dt $$

According to Eq. (1) and the longest discharging time of capacitors in Fig. 3, it is possible to calculate the discharge value of capacitors *C*_*1*_ to *C*_*3*_ for a pure resistive load, which is the worst loading condition through Eqs. (2) to (4).

2 $$ Q_{C1} = 2\int_{{t_{8} }}^{T/4} {I_{P} } \sin \left( {2\pi f_{o} t} \right)dt $$

3 $$ Q_{C2} = 2\int_{{t_{7} }}^{T/4} {I_{P} } \sin \left( {2\pi f_{o} t} \right)dt $$

4 $$ Q_{C3} = 2\int_{{t_{4} }}^{T/4} {I_{P} } \sin \left( {2\pi f_{o} t} \right)dt $$

In Fig. 3, the output voltage generation pattern based on the fundamental frequency switching technique is illustrated, which can divide the half-cycle period (*T/2*) into 16 time steps. These time steps are calculated by Eq. (5).

5 $$\begin{aligned} t_{1} & = \frac{{\sin^{ - 1} \left( 1/16 \right)}} {2\pi f},t_{2} = \frac{{\sin^{ - 1} \left( 3/16 \right)}} {2\pi f},t_{3} = \frac{{\sin^{ - 1} \left( 5/16 \right)}} {2\pi f}   \\ t_{4} & = \frac{{\sin^{ - 1} \left( 7/16 \right)}} {2\pi f},t_{5} = \frac{{\sin^{ - 1} \left(9/16 \right)}} {2\pi f},t_{6} = \frac{{\sin^{ - 1} \left(11/16 \right)}} {2\pi f}  \\ t_{7} & = \frac{{\sin^{ - 1} \left( 13/16 \right)}} {2\pi f},t_{8} = \frac{{\sin^{ - 1} \left(15/16 \right)}} {2\pi f} \end{aligned}$$

If the allowable voltage ripple of capacitors (*V*_*R*_) is considered between 5 and 10%, the capacity of the capacitors can be calculated by the following equation.

6 $$ {\text{C}} \ge \frac{{{\text{Q}}_{{\text{C}}} }}{{\% {\text{VR}} \times {\text{V}}_{C} \times 2\pi f}} $$

## Inverter losses evaluation

In general, losses of switched-capacitor multi-level inverters are divided into three parts, which are studied in this section. The switched-capacitor multi-level inverter power loss is according to Eq. (7), where P_S_ is the switching loss, P_C_ is the conduction loss, and P_Ripple_ is the ripple loss. The switching and conduction losses are related to power semiconductor equipment, and the ripple losses are related to voltage drop and voltage ripple of capacitors^19,20^.

7 $$ P_{losses} = P_{S} + P_{C} + P_{Ripple} $$

### Switching losses

Switching losses occur due to delays in the conduction behavior of the switches. Since the switches dissipate energy both during the on-state and off-state, the energy loss of a semiconductor switch can be calculated using Eq. (8). In this equation, N_ON_ and E_ON_ represent the number of times the switch turns on and the energy dissipated during the on-state, respectively. While N_OFF_ and E_OFF_ represent the number of times the switch turns off and the energy dissipated during the off-state, respectively.

8 $$ P_{S} = f\left( {N_{ON} E_{ON} + N_{OFF} E_{OFF} } \right) $$

### Conduction losses

**Figure 4:**
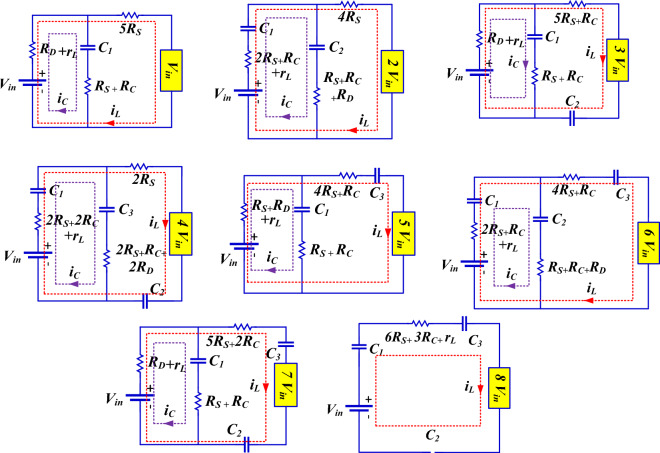
Equivalent circuit of conduction losses of the proposed structure.

The internal resistance of the semiconductor devices that are in the path of current constitutes the conduction losses. The equivalent circuit of conduction losses for the proposed structure is shown in Fig. 4 based on the output voltage levels. By applying KVL (Kirchhoff's Voltage Law) in these circuits, the conduction losses for each output voltage level can be calculated using the equations provided. It should be noted that, the charge current is calculated using the equations provided in Table 2.

9 $$ P_{{C_{1} }} = \left( {i_{C} + i_{L} } \right)^{2} \left( {R_{D} + r_{L} } \right) + \left( {R_{S} + R_{C} } \right)i_{C}^{2} + \left( {5R_{S} } \right)i_{L}^{2} $$

10 $$ P_{{C_{2} }} = \left( {i_{C} + i_{L} } \right)^{2} \left( {2R_{S} + R_{C} + r_{L} } \right) + \left( {R_{S} + R_{C} + R_{D} } \right)i_{C}^{2} + \left( {4R_{S} } \right)i_{L}^{2} $$

11 $$ P_{{C_{3} }} = \left( {i_{C} + i_{L} } \right)^{2} \left( {R_{D} + r_{L} } \right) + \left( {R_{S} + R_{C} } \right)i_{C}^{2} + \left( {5R_{S} + R_{C} } \right)i_{L}^{2} $$

12 $$ P_{{C_{4} }} = \left( {i_{C} + i_{L} } \right)^{2} \left( {2R_{S} + 2R_{D} + r_{L} } \right) + \left( {2R_{S} + R_{C} + 2R_{D} } \right)i_{C}^{2} + \left( {2R_{S} } \right)i_{L}^{2} $$

13 $$ P_{{C_{5} }} = \left( {i_{C} + i_{L} } \right)^{2} \left( {R_{S} + R_{D} + r_{L} } \right) + \left( {R_{S} + R_{C} } \right)i_{C}^{2} + \left( {4R_{S} + R_{C} } \right)i_{L}^{2} $$

14 $$ P_{{C_{6} }} = \left( {i_{C} + i_{L} } \right)^{2} \left( {2R_{S} + R_{C} + r_{L} } \right) + \left( {R_{S} + R_{C} + R_{D} } \right)i_{C}^{2} + \left( {4R_{S} + R_{C} } \right)i_{L}^{2} $$

15 $$ P_{{C_{7} }} = \left( {i_{C} + i_{L} } \right)^{2} \left( {R_{D} + r_{L} } \right) + \left( {R_{S} + R_{C} } \right)i_{C}^{2} + \left( {5R_{S} + 2R_{C} } \right)i_{L}^{2} $$

16 $$ P_{{C_{8} }} = \left( {6R_{S} + 3R_{C} + r_{L} } \right)i_{L}^{2} $$

**Table 2:** Calculation of charging current based on voltage levels.

Output voltage	Charging current (i_C_)
V_in_	$i_{C} = \frac{{V_{in} - V_{D} - V_{C1} }}{{R_{S} + R_{C} + R_{D} }},i_{L} = \frac{{V_{in} }}{{R_{L} }}$
2V_in_	$i_{C} = \frac{{V_{in} + V_{C1} - V_{D} - V_{C2} }}{{3R_{S} + 2R_{C} + R_{D} }},i_{L} = \frac{{2V_{in} }}{{R_{L} }}$
3V_in_	$i_{C} = \frac{{V_{in} - V_{D} - V_{C1} - i_{L} .R_{D} }}{{R_{S} + R_{C} + R_{D} }},i_{L} = \frac{{3V_{in} }}{{R_{L} }}$
4V_in_	$i_{C} = \frac{{V_{in} + V_{C1} + V_{C2} - V_{C3} }}{{4R_{S} + 3R_{C} + 2R_{D} }},i_{L} = \frac{{4V_{in} }}{{R_{L} }}$
5V_in_	$i_{C} = \frac{{V_{in} - V_{D} - V_{C1} - i_{L} \left( {R_{S} + R_{D} } \right)}}{{2R_{S} + R_{C} + R_{D} }},i_{L} = \frac{{5V_{in} }}{{R_{L} }}$
6V_in_	$i_{C} = \frac{{V_{in} + V_{C1} - V_{D} - V_{C2} - i_{L} \left( {2R_{S} + R_{C} } \right)}}{{3R_{S} + 2R_{C} + R_{D} }},i_{L} = \frac{{6V_{in} }}{{R_{L} }}$
7V_in_	$i_{C} = \frac{{V_{in} - V_{D} - V_{C1} - i_{L} .R_{D} }}{{R_{S} + R_{C} + R_{D} }},i_{L} = \frac{{7V_{in} }}{{R_{L} }}$
8V_in_	$i_{C} = 0,i_{L} = \frac{{8V_{in} }}{{R_{L} }}$

Over a complete cycle, the instantaneous conduction losses are averaged, and the average conduction losses for each output voltage level are presented in Table 3. By calculating the average conduction losses separately for each level, the total conduction losses can be calculated using the following equation:

17 $$ P_{{C_{total} }} = \sum\limits_{n = 1}^{8} {\left( {P_{AC} } \right)} n $$

**Table 3:** Average conduction losses at each voltage level.

$P_{{AC_{1} }} = 4 \times \frac{{\theta_{2} - \theta_{1} }}{2\pi } \times \left( {P_{{C_{1} }} } \right)$	$P_{{AC_{2} }} = 4 \times \frac{{\theta_{3} - \theta_{2} }}{2\pi } \times \left( {P_{{C_{2} }} } \right)$
$P_{{AC_{3} }} = 4 \times \frac{{\theta_{4} - \theta_{3} }}{2\pi } \times \left( {P_{{C_{3} }} } \right)$	$P_{{AC_{4} }} = 4 \times \frac{{\theta_{5} - \theta_{4} }}{2\pi } \times \left( {P_{{C_{4} }} } \right)$
$P_{{AC_{5} }} = 4 \times \frac{{\theta_{6} - \theta_{5} }}{2\pi } \times \left( {P_{{C_{5} }} } \right)$	$P_{{AC_{6} }} = 4 \times \frac{{\theta_{7} - \theta_{6} }}{2\pi } \times \left( {P_{{C_{6} }} } \right)$
$P_{{AC_{7} }} = 4 \times \frac{{\theta_{8} - \theta_{7} }}{2\pi } \times \left( {P_{{C_{7} }} } \right)$	$P_{{AC_{8} }} = 4 \times \frac{{{\pi /2} - \theta_{8} }}{2\pi } \times \left( {P_{{C_{8} }} } \right)$

### Ripple loss

The energy lost when the capacitor is charged through the source is called ripple loss, which is affected by load characteristics and switching frequency. The ripple losses can be calculated using Eq. (18). In this equation, capacitor voltage ripple *ΔV*_*C*_ is calculated through Eqs. (19) to (21).

18 $$ P_{Ripple} = 2f\sum\limits_{i = 1}^{{N_{C} }} \frac{1}{2} C_{i} \left( {\Delta V_{Ci} } \right)^{2} $$

19 $$ \Delta V_{C1} = \frac{{I_{o} }}{{\pi fC_{1} }} \times \left[ {\cos \left( {2\pi ft_{8} - \varphi } \right) - \sin \varphi } \right] $$

20 $$ \Delta V_{C2} = \frac{{I_{o} }}{{\pi fC_{2} }} \times \left[ {\cos \left( {2\pi ft_{7} - \varphi } \right) - \sin \varphi } \right] $$

21 $$ \Delta V_{C3} = \frac{{I_{o} }}{{\pi fC_{3} }} \times \left[ {\cos \left( {2\pi ft_{4} - \varphi } \right) - \sin \varphi } \right] $$

**Figure 5:**
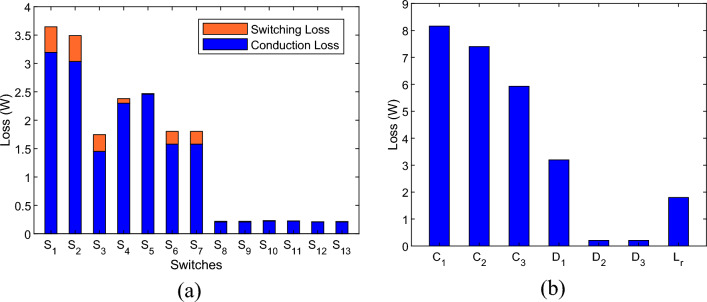
Losses of the proposed inverter, (**a**) switching and conduction losses of the switches, (**b**) conduction losses of the diodes, inductor, and ripple losses of the capacitors.

Using the provided equations, the losses of the proposed inverter can be calculated according to Fig. 5. The calculations are performed under the conditions where the input voltage is 100 V, the output voltage is 800 V, the output current is 2.8 amperes, a resistive-inductive load with an impedance of 250 ohms, 80 mH, and the output power is 1173 watts. The loss calculations were performed using the information provided in the datasheets for the IGBT switch IKFW60N60DH3E (600V/50A) and the diode FFPF30U60S (600V/30A). The energy loss curves (E_OFF_, E_ON_) relative to the collector-emitter voltage of the switch are provided by the manufacturer, and these curves are used in simulating losses.

## Comparative evaluation

To more accurately examine the advantages and disadvantages of the proposed structure, a comprehensive comparison with other 17-level switched-capacitor structures in terms of the number of voltage sources (*N*_*dc*_), the number of power switches (*N*_*S*_), the number of drivers (*N*_*dr*_), the number of capacitors (*N*_*C*_), the number of diodes (*N*_*D*_), the maximum number of on power switches in the load path (*N*_*ms*_), the maximum standing voltage (MSV) of the power switches, the total standing voltage (TSV) of all the power switches, the boost factor (BF), and the cost factor (CF) are provided in Table 4. In this table, the cost factor is presented based on the ratio of the number of devices per the output levels and is calculated by Eq. (22)^21^. In this equation, α is a weighting factor that can be considered 0.5 or 1.5^5^. The weighting factor is multiplied by TSV to calculate the cost factor under fair conditions. If the priority of design and comparison is with the number of circuit elements, this parameter is considered 0.5, and if the priority of design and comparison is with blocking voltage of equipment, this parameter is considered 1.5. Common CHB, NPC, and FC structures require many components to generate 17 voltage levels. This issue has caused them to have a high cost factor and are not cost-effective for various applications. The structures presented in^22,23,25^, and^27^ are not single-source and produce 17-level voltage in the case of asymmetric sources. The main challenge of these structures is how to provide DC voltage sources. According to Table 4, the TSV of the proposed structure is lower than compared to single-source 17-level structures. Additionally, the structures presented in^25,27^, and^28^ need switches with a high-rated voltage because the maximum voltage that can be tolerated by some switches in these structures is eight times the input voltage, and this issue can limit their application. At the same time, the MSV of the propsosed structure is half of the maximum output voltage. This feature improves the application of the proposed structure with lower reated voltage swtches, which will naturally require less cost. According to Table 4, the proposed 17-level switched capacitor structure introduces lower cost factor in comparison wth other similar recent presented 17-level structures for both 0.5 and 1.5 weighting factors. Also, the maximum number of conductiong power switches in the load path is suitable count compared to other similar 17-level structures.

**Table 4:** Comparing the proposed structure with other 17-level switched-capacitor structures.

Topology	N_dc_	N_S_	N_dr_	N_C_	N_D_	TC	N_ms_	TSV_pu_	MSV	BF	CF	
											= 0.5α	= 1.5α
CHB	8	32	32	0	0	72	32	32	1	1	37.64	52.70
NPC	2	32	32	16	32	114	16	32	1	1	15.05	18.82
FC	2	32	32	16	32	114	16	32	1	1	15.05	18.82
^12^	1	13	13	3	3	33	7	5.625	4	8	2.04	2.37
^15^	1	12	12	4	5	34	6	6.25	4	8	2.12	2.49
^22^	2	10	8	2	0	22	5	4.5	2	4	2.61	3.14
^23^	2	12	11	3	1	29	7	4.5	5	1.6	3.44	3.97
^24^	1	14	14	3	2	34	8	5.625	4	8	2.10	2.43
^25^	3	11	11	1	0	26	5	4.875	8	1	4.48	5.34
^26^	1	14	14	3	2	34	7	6.25	4	8	2.12	2.49
^27^	2	18	18	6	2	46	9	7	8	4	5.58	6.41
^28^	1	16	14	4	0	35	8	6.5	8	8	2.19	2.57
^29^	4	10	9	0	0	23	4	2	7	1	4.70	5.17
^30^	1	19	18	0	4	42	12	3.625	2	4	2.51	2.73
^31^	2	10	10	1	1	24	5	5.28	7	1.14	2.89	3.52
Proposed	1	13	13	3	3	33	7	5.625	4	8	2.04	2.37

**Figure 6:**
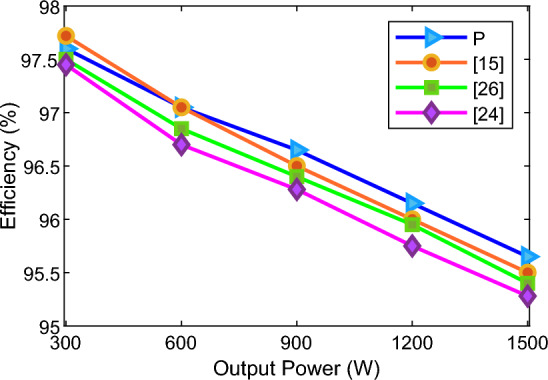
Comparison diagram of the proposed structure efficiency with other structures.

Figure 6 illustrates the efficiency comparison of the proposed structure with other structures for different output powers. The switching pattern is effective in the efficiency of the converters. The structures controlled by low-frequency pattrens such as Nearest Level Control (NLC) or Selective Harmonic Elimination (SHE) strategy have fewer losses, and as a result, show higher efficiency. In this comparison, the efficiency of all structures has been evaluated based on the NLC modulation scheme to achieve a fair and just comparison. One of the critical parameters in the total losses of the multilevel inverters is the number of switches in ON mode at different voltage levels. The lower the number of ON switches in the load and capacitor charging paths, the lower the conduction losses, and as a result, the total losses of the inverter are decreased. Based on the comparison Table, multi-source structures have fewer ON switches in producing voltage levels due to the utilization of more DC sources. In the proposed structure, a maximum of six switches are placed in the path of the output load current, which is the lowest value for single-source structures. Therefore, the conduction losses of the proposed structure can be lower than those of similar structures based on the number of conducting switches in different voltage levels. Following Fig. 6, the efficiency of the structures decreases to some extent with the increase of the output power. The structure of^15^ is more efficient than the proposed structure at low powers with a tiny difference. However, by increasing the output power, the efficiency of the proposed structure is more than the efficiency of the structure in^15^. According to this figure, the efficiency of the proposed structure is equal to or better than that of the comparative structures. All the comparative structures in this section are of the 17-level single-source switched-capacitor inverter type presented recently.

22 $$ C_{f} = \frac{{\left( {N_{S} + N_{dr} + N_{C} + N_{D} + \alpha TSV_{pu} } \right) \times N_{dc} }}{{N_{l} }} $$

## Simulation and laboratory results

**Figure 7:**
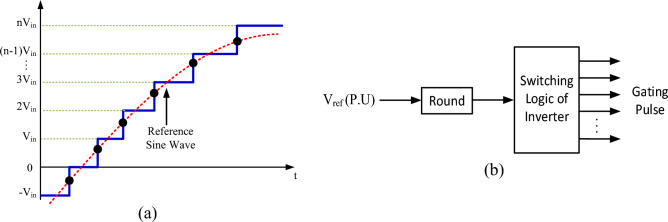
(**a**) Nearest level control modulation, (**b**) the method of implementing the nearest level control modulation.

**Figure 8:**
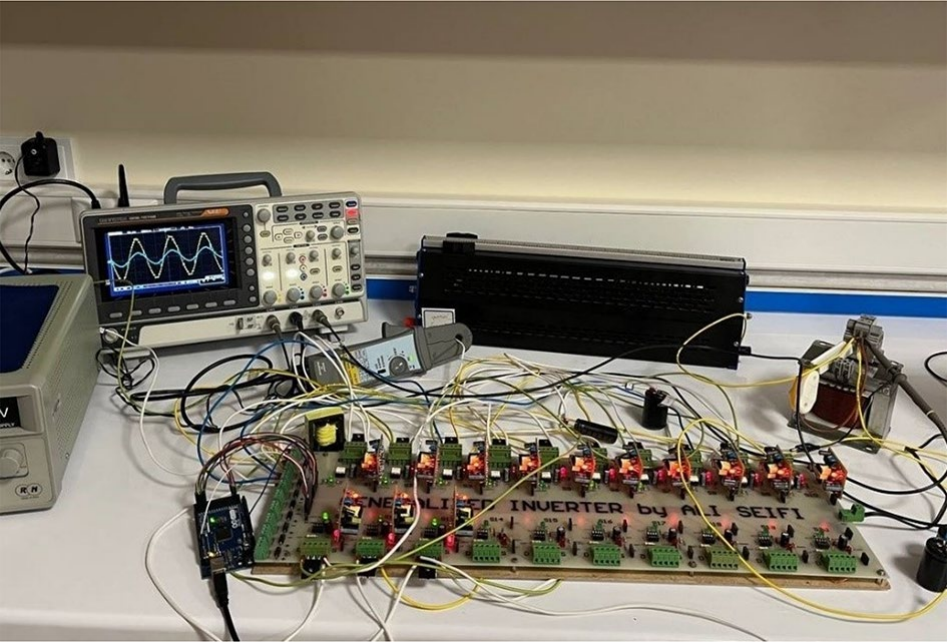
Implementation schematic of the proposed structure.

In this section, simulation and experimental results have been presented to check the correctness of the proposed circuit's performance, as well as the analyses illustrated in the previous sections. Nearest Level Control (NLC) switching scheme has been used to control the power switches of the proposed structure. In this method, the nearest voltage level traces the sinusoidal voltage waveform, and thus, voltage levels are created. Figure 7 displays the implemented switching scheme. The results of simulation and implementation are demonstrated under various conditions including pure resistive as well as resistive-inductive loads, dynamic change in load, modulation index, and output frequency. Moreover, the input voltage and current waveform, the voltage waveform of the switches to show their voltage stress, and the voltage and current waveform of the capacitors to check the voltage and current stress of the capacitors and the switches on the capacitors charging path are displayed as well. The parameters used for simulating and implementing the proposed structure are given in Table 5. The circuit diagram of the proposed structure implemented in the laboratory environment is shown in Fig. 8. The proposed inverter prototype was tested and evaluated with an output power of 175 W for a resistive-inductive load and an output power of 245 W for a purely resistive load.

**Table 5:** Simulation and implementation parameters information.

Parameters	Values
Input voltage	23 V
Output frequency	50 Hz
C_1_	1500 µF
C_2_	2200 µF
C_3_	2700 µF
R-L load	80Ω–120 mH
Mosfet	MOSTET IRFP450
Driver	TLP250
Microcontroller	Arduino Mega 2560
Diode	MUR1560

**Figure 9:**
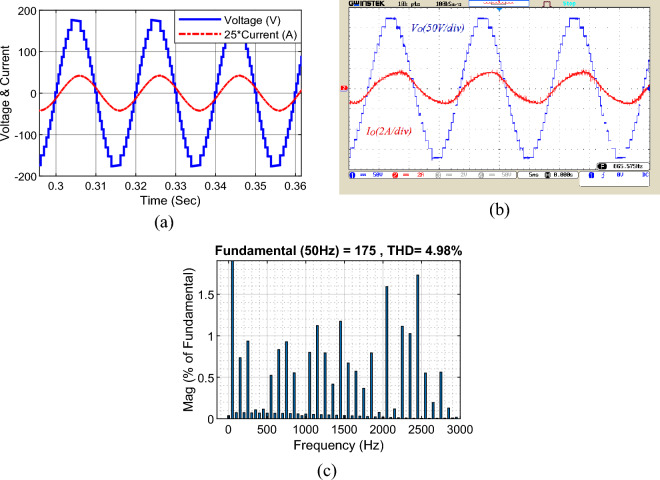
Output voltage and current waveform under resistive-inductive load, (**a**) simulation, (**b**) laboratory, (**c**) THD of the output voltage.

Figure 9 displays the output voltage and current under resistive-inductive load (*Z*_*l*_ = 80 Ω + 120 mH). Figure 9a shows the results of the simulation, according to which the output voltage is stepped and pseudo-sinusoidal and has 17 voltage levels. The peak output voltage is 175 V, and the peak output current is about 2 A. According to this figure, the capability to increase the output voltage by 8 times is confirmed compared to the input voltage of 23 V. Due to the low voltage ripple of the capacitors, the steps of different voltage levels are equal to 23 V. Figure 9b is related to the results of implementing the output voltage and current in similar conditions. In Fig. 9c the harmonic distortion of the output voltage is shown that the THD of the 17-level output voltage is 4.98%.

**Figure 10:**
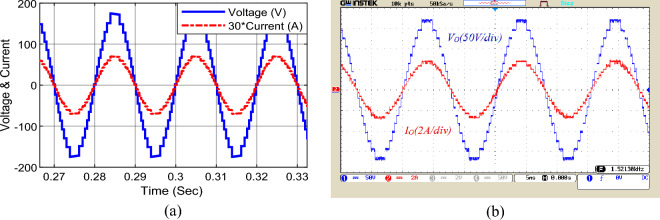
Voltage waveform and output current under pure resistive load; (**a**) simulation, (**b**) laboratory.

Figure 10 displays the output voltage and current waveform under pure resistive load (*Z*_*l*_ = 80 Ω). In this condition, the peak output current reaches about 2.8 A. In this figure, the voltage steps at different levels are equal, which indicates the proper voltage regulation of the capacitors. The agreement of simulation results and laboratory implementation can be seen in this figure.

**Figure 11:**
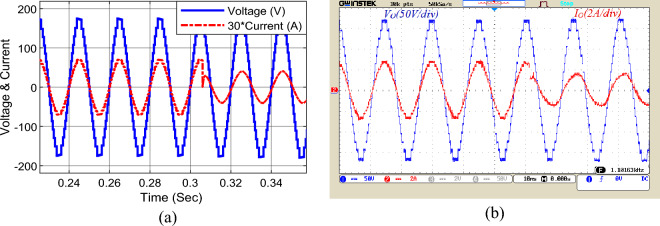
The output load dynamic change, (**a**) simulation, (**b**) laboratory.

To evaluate the performance of the inverter in dynamic conditions, load dynamic change has been assessed in Fig. 11. In this figure, the performance of the proposed structure is shown for the sudden change in output load at t = 0.305 s from the pure resistive load of Z_l_ = 90 Ω to the resistive-conductive load of *Z*_*l*_ = 80 Ω + 120 mH. According to this figure, the 17-level waveform of the output voltage does not change under the sudden load change condition. The proposed inverter can adequately feed different loads with different power factors, and under load changes as well.

**Figure 12:**
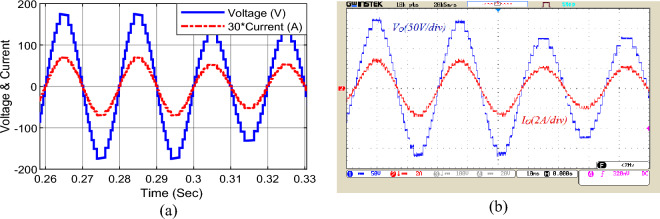
Dynamic change in modulation index, (**a**) simulation, (**b**) laboratory.

Figure 12 presents the output voltage and current under sudden change of modulation index. According to this figure, by changing the modulation index from 1 to 0.7 at t = 0.3 s, the output voltage waveform reaches from 17 to 13 levels. In addition, the output voltage and current will change from 175 to 130 V and from 2.4 A to 2 A, respectively.

**Figure 13:**
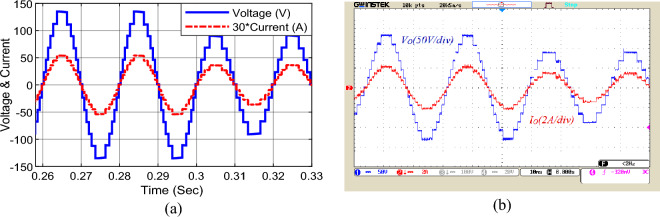
The second mode dynamic change in modulation index; (**a**) simulation, (**b**) laboratory.

Figure 13 demonstrates another mode of modulation index change. In this case, when the modulation index is changed from 0.7 to 0.5, the 13-level output voltage with a peak of 130 V changes to a 9-level voltage with a peak of 90 V. It is apparent that the proposed structure can correctly create output levels in both cases of dynamic change in modulation index.

**Figure 14:**
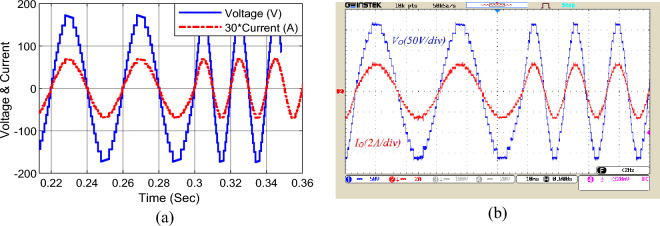
The output frequency change; (**a**) simulation, (**b**) laboratory.

**Figure 15:**
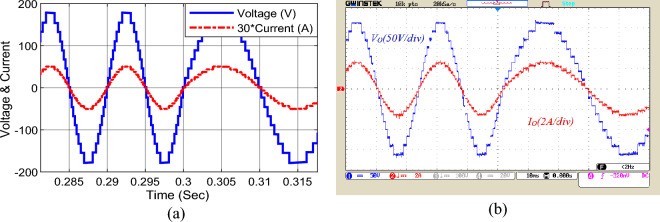
The second mode of the output frequency changes, (**a**) simulation, (**b**) laboratory.

In Figs. 14 and 15, the dynamic response of the proposed structure to the output frequency change is demonstrated. In Fig. 14, the output frequency has been changed from 50 to 25 Hz, and in Fig. 15, the output frequency has been changed from 50 o 100 Hz. According to these two figures, the proposed structure can feed the output load at different frequencies, and the conditions of dynamic frequency change as well.

**Figure 16:**
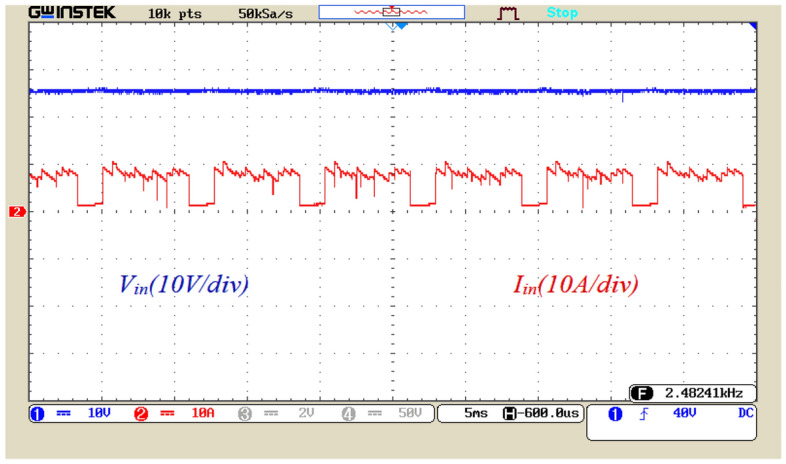
Input voltage and current waveform.

Figure 16 displays the voltage and current waveform of the input power supply. The DC value of the input voltage is equal to 23 V, and the peak input current is less than 10 A. Employing soft charging results in drawing suitable limited currents from the input source to charge the capacitors. According to Fig. 16, there is no problem of inrush current to charge the capacitors in the proposed structure.

**Figure 17:**
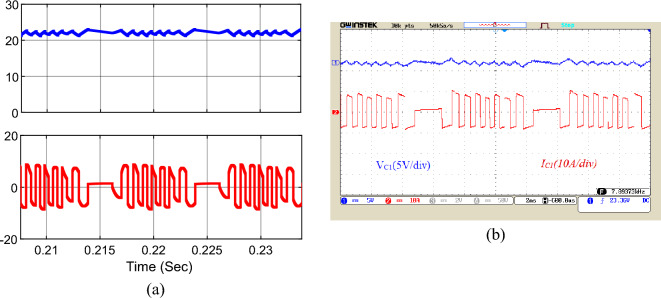
Voltage and current waveforms of the capacitor *C*_*1*_; (**a**) simulation, (**b**) laboratory.

**Figure 18:**
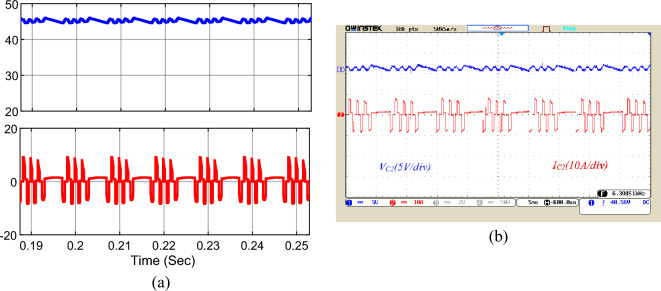
Voltage and current waveforms of the capacitor *C*_*2*_; (**a**) simulation, (**b**) laboratory.

**Figure 19:**
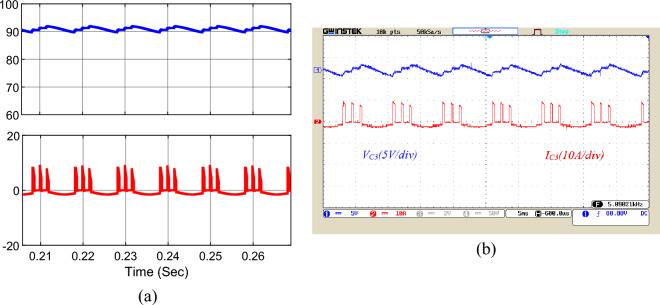
Voltage and current waveforms of the capacitor *C*_*3*_; (**a**) simulation, (**b**) laboratory.

The simulation and implementation results for the voltage and current waveforms of capacitors *C*_*1*_ to *C*_*3*_ are shown in Figs. 17, 18, and 19, respectively. According to these figures, the voltage of capacitor *C*_*1*_ is 23 V, the voltage of capacitor *C*_*2*_ is 46 V, and the voltage of capacitor *C*_*3*_ is around 92 V. Choosing the correct capacity for the capacitors has made the voltage ripple of the capacitors within the standard range and less than 10%, which has a significant influence on reducing the ripple losses of the converter. Besides, the peak current of the capacitors is about 10 A. Utilizing the soft charging in the proposed structure has led to drawing no unusual currents to charge the capacitors, and there is no challenge of inrush currents to charge the capacitors.

According to Fig. 3, capacitor C_3_ has a rated voltage of 4V_in_ and a long discharge time. According to relation (6), which is related to the calculation of capacitance, the nominal voltage of the capacitor is in the denominator of this equation. Since the voltage of this capacitor is 4 times the input voltage, a large capacitance is not obtained for this capacitor despite a relatively long discharge time. In other words, the long discharge time in the numerator of the capacitance equation becomes ineffective by the high-rated voltage in the denominator.

**Figure 20:**
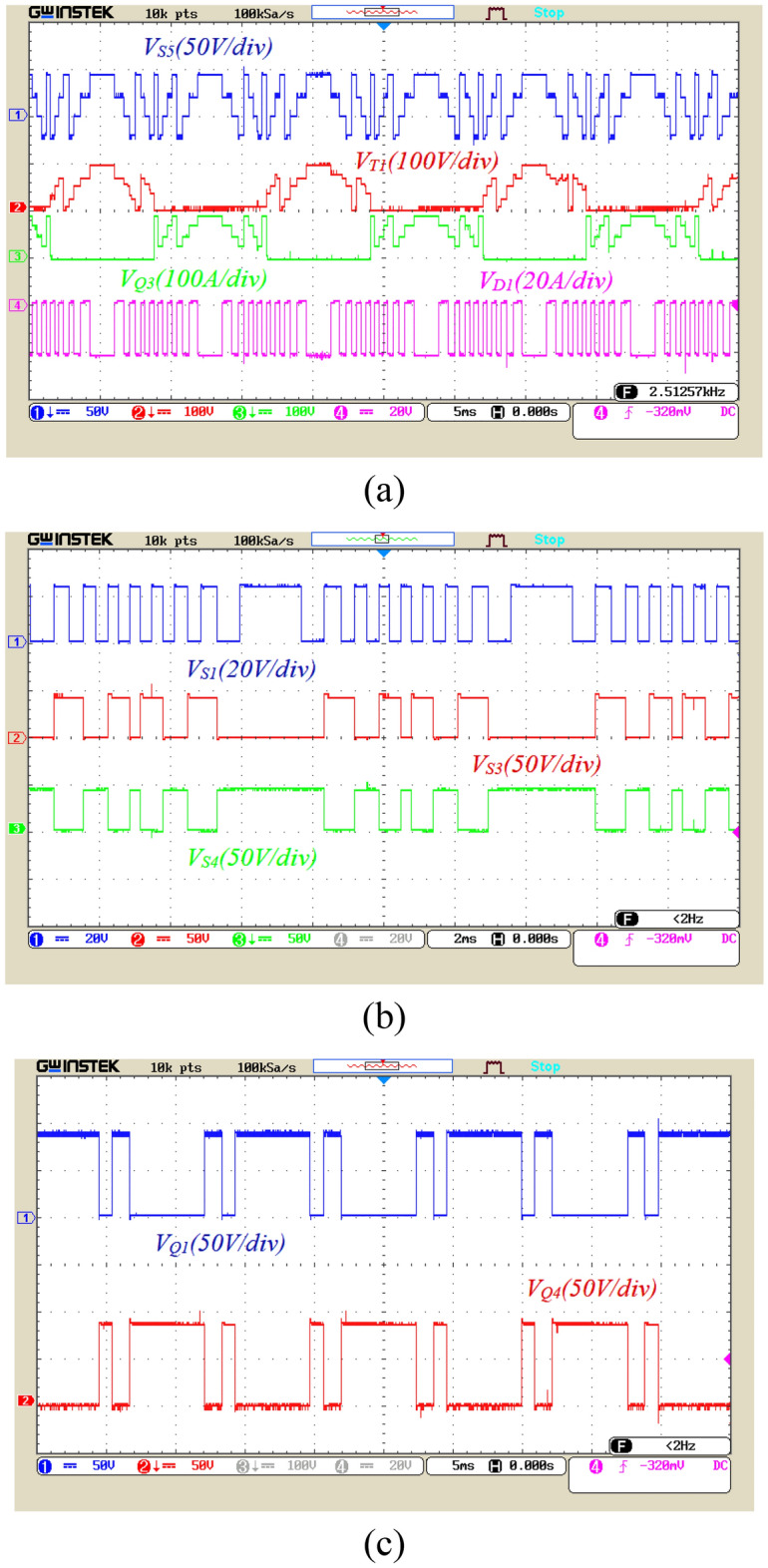
The voltage waveform of the two ends of the switches; (**a**) the voltage between the ends of the switches *S*_*5*_, *T*_*1*_, *Q*_*3*_, and diode *D*_*1*_ (**b**) the voltage between the ends of the switches *S*_*1*_, *S*_*3*_, *S*_*4*_, and (**c**) the voltage between the ends of the switches *Q*_*1*_, and *Q*_*4*_*.*

Figure 20 displays the voltage across the switches and power diodes. Figure 20a is related to the voltage stress of power switches *S*_*5*_, *T*_*1*_, *Q*_*3*_, and diode *D*_*1*_. According to this figure, the voltage stress of the mentioned power switches is 25%, 50%, and 50% of the peak output voltage, respectively, and the voltage of diode *D*_*1*_ is 12.5% of the peak output voltage. Figure 20b presents the voltage waveform of power switches *S*_*1*_, *S*_*3*_, *S*_*4*_. According to this figure, the voltage stress of the mentioned switches is 12.5%, 25%, and 25% of the peak output voltage, respectively. Figure 20c also illustrates the voltage stress of power switches *Q*_*1*_ and *Q*_*4*_, which are 50% of the output voltage. Following the results of these figures, the maximum voltage stress of the power switches is half of the output voltage in the proposed structure, which is considered a significant advantage for this structure. The nominal voltage of these switches can be selected as half of the nominal voltage of the inverter, reducing the cost of power electronic devices in the proposed structure.

## Conclusion

In this paper, a 17-level single-source structure with octuple voltage gain is proposed. Because the H-bridge module is not utilized in this structure, none of the switches must withstand the maximum output voltage. The maximum blocked voltage of some switches is 50% of the output voltage, and the rest of the switches are less than this value, which makes it possible to employ switches with a nominal voltage lower than the output voltage. To control the charging current of the capacitors, a current limiting inductor is used in the input source path, and the charging current of the capacitors is well limited. Based on the comparison conducted, the proposed structure has the lowest cost factor among the compared structures, with a value of 2.04. The THD of the output voltage is 4.98%. The efficiency of the proposed structure in the output power of 1500 W is more than 95%.

## Data Availability

All data generated and analysed during the current study are available from the corresponding author on reasonable request.
